# Urinary sodium wasting and disrupted collecting duct function in mice with distal renal tubular acidosis mutations

**DOI:** 10.1242/dmm.052138

**Published:** 2025-05-23

**Authors:** Priyanka Mungara, Kristina MacNaughton, A. K. M. Shahid Ullah, Grace Essuman, Forough Chelangarimiyandoab, Rizwan Mumtaz, J. Christopher Hennings, Christian A. Hübner, Dominique Eladari, R. Todd Alexander, Emmanuelle Cordat

**Affiliations:** ^1^ Department of Physiology, University of Alberta, Edmonton, AB T6G2H7, Canada; ^2^ Membrane Protein Disease Research Group, University of Alberta, Edmonton, AB T6G2H7, Canada; ^3^ Institute of Human Genetics, Jena University Hospital, Friedrich Schiller University, 07747 Jena, Germany; ^4^ Center for Rare Diseases, Jena University Hospital, Friedrich Schiller University, 07747 Jena, Germany; ^5^ Service de Médecine de Précision des Maladies Rénales et Métaboliques, CHU Amiens Picardie, Faculté de Médecine d'Amiens, Université de Picardie Jules Verne, 80054 Amiens, France; ^6^ Institut National de la Santé et de la Recherche Médicale U970, Paris centre de recherche cardiovasculaire (PARCC), 75015 Paris, France; ^7^ French Clinical Research Infrastructure Network (F-CRIN), Investigation Network Initiative Cardiovascular and Renal Clinical Trialist (INI-CRCT), 54500 Vandœuvre-lès-Nancy, France; ^8^ Department of Pediatrics, University of Alberta, Edmonton, AB T6G2H7, Canada

**Keywords:** Distal renal tubular acidosis, Urinary sodium waste, Intercalated cells, Collecting duct, Bicarbonate transporter, Acid-base balance

## Abstract

Distal renal tubular acidosis (dRTA) results in metabolic acidosis owing to impaired urinary acidification and can result in an unexplained urinary sodium-wasting phenotype. We report the generation and characterization of a novel dRTA mutant mouse line, *Ae1* L919X knock-in (KI). Homozygous L919X KI mice exhibit typical dRTA features, including reduced ability to acidify urine in response to an acid load. This renal acidification defect was associated with a reduced number of kAE1-positive type A intercalated cells. To assess whether these mice exhibit urinary sodium wasting, homozygous L919X KI mice and the previously described R607H KI mice were fed a salt-depleted acid diet. In line with human patients, both mouse strains exhibited urinary sodium loss. Additionally, we identified increased expression of tight junction proteins claudin 4 and claudin 10b, suggesting a compensatory paracellular pathway. Consistent with data from human patients, L919X KI mice displayed a milder phenotype than that of R607H KI mice. Our findings reveal that both mouse strains are appropriate models for dRTA with a urinary salt-wasting phenotype and compensatory upregulation of the paracellular pathway.

## Teaser

**Summary:** Mouse lines carrying *Ae1* L919X or R607H knock-in variations that mimic human distal renal tubular acidosis (dRTA) display urinary sodium waste, as seen in some patients with dRTA, and upregulation of tight junction proteins claudin 4 and 10b, when exposed to a salt-depleted acid challenge.

## INTRODUCTION

Renal tubular acidosis is a group of disorders in which dysregulation of acid-base homeostasis occurs owing to improper bicarbonate reabsorption or non-volatile acid excretion by the nephron (Mohebbi and Wagner, 2018; Alper, 2010; Batlle et al., 2001; Giglio et al., 2021; Wagner et al., 2023). Inherited or acquired distal renal tubular acidosis (dRTA) impairs distal proton secretion and, consequently, renal acid excretion (Alper, 2010; Giglio et al., 2021; Fry and Karet, 2007; Karet, 2002; Laing et al., 2005; Unwin and Capasso, 2001). In addition to alkaline urine, patients with dRTA can present with hyperchloremic metabolic acidosis (Alper, 2010; Wagner et al., 2023; Fry and Karet, 2007; Karet, 2002; Laing et al., 2005; Unwin and Capasso, 2001; Alonso-Varela et al., 2020), hypokalemia (Aronson and Giebisch, 2011), nephrocalcinosis/kidney stones (Alonso-Varela et al., 2020) and renal sodium wasting (Sebastian et al., 1976) with urinary-concentrating defects (Wagner et al., 2023). Notably, in some patients with inherited dRTA, correction of the metabolic acidosis does not prevent renal sodium wasting and impaired renal salt handling, consistent with an ion tubular nephropathy (Sebastian et al., 1976).

dRTA affects the collecting duct (CD), a primary regulator of acid-base homeostasis and electrolyte and fluid status, which finetunes the final urine composition (Fry and Karet, 2007). The CD is composed of principal cells (PCs) and intercalated cells (ICs). PCs mediate electrogenic salt and water reabsorption via the epithelial sodium channel (ENaC; also known as SCNN1) and aquaporin 2 (AQP2) (Lashhab et al., 2019a; Roy et al., 2015). Type A (A-IC) and type B (B-IC) ICs regulate acid-base homeostasis (Saxena et al., 2021). A-ICs secrete protons via an apical vacuolar proton ATPase (V-ATPase) and promote reabsorption of bicarbonate via the basolateral kidney anion exchanger 1 (kAE1; encoded by *Slc4a1*, referred to here as *Ae1*). B-ICs facilitate bicarbonate secretion through the apical chloride/bicarbonate exchanger pendrin and reabsorb protons via a basolateral V-ATPase. They also contribute to electroneutral salt reabsorption through the coupled activity of pendrin and the sodium-dependent chloride/bicarbonate exchanger (NDCBE; also known as SLC4A8) (Purkerson et al., 2014; Peña-Münzenmayer et al., 2016; Wall et al., 2020). At least one other intercalated cell type exists – non-A, non-B ICs, which are characterized by apical expression of both pendrin and the V-ATPase. The role of non-A, non-B ICs is unclear.

Inherited dRTA may arise from autosomal dominant or recessive mutations in the *SLC4A1* gene encoding kAE1 (Bruce et al., 1997, 2000; Karet et al., 1998; Jarolim et al., 1998). One such dominant variant is *AE1* R589H, which is located on the cytosolic side of transmembrane domain 6 in the gate domain of kAE1 (Arakawa et al., 2015; Zhekova et al., 2022; Reithmeier et al., 2016). *In vitro* studies have revealed retention in the endoplasmic reticulum (Jarolim et al., 1998; Cordat et al., 2006) and apical mistargeting (Toye et al., 2004) of the mutant protein, and a 20-50% reduction in its anion exchange activity compared to wild-type (WT) kAE1 (Jarolim et al., 1998). However, the mutant protein is able to hetero-oligomerize with WT kAE1 (Cordat et al., 2006; Quilty et al., 2002), thereby resulting in slightly reduced chloride/bicarbonate exchange at the cell surface. *Ae1* R901X is another common autosomal dominant mutation, which causes a cytosolic C-terminal tail truncation. In *in vitro* studies, the R901X mutant has normal transport activity, but is retained intracellularly and/or apically mistargeted (Cordat et al., 2006, 2003; Quilty et al., 2002; Toye et al., 2002), similarly to the R589H mutant. However, *in vivo* data from transgenic mice (Mumtaz et al., 2017; Stehberger et al., 2007) and kidney biopsies from patients with dRTA (DeFranco et al., 1995; Vichot et al., 2017) indicate that dRTA-causing variants of kAE1 decrease the abundance of kAE1-positive ICs, emphasizing the limitations of *in vitro* approaches. The *Ae1* R607H knock-in (KI) mouse model (the R607H mutation is orthologous to the human dRTA R589H mutation) recapitulates classical metabolic acidosis (Mumtaz et al., 2017). In this model, R607H KI/KI mice exhibited proper kAE1 basolateral membrane targeting but decreased staining intensity for both kAE1 and the B1 subunit of V-ATPase, and decreased *Ae1* mRNA and protein abundance. In addition, the remaining ICs of these mutant mice were larger in size and accumulated the autophagy marker p62 (also known as SQSTM1). Together, these results are consistent with a reduced number of kAE1-positive A-ICs in R607H KI/KI mice (Mumtaz et al., 2017).

To date, the R607H KI mouse is the only dominant mouse model available. In the current work, we report the generation and characterization of a second dRTA mutant mouse model, carrying the autosomal dominant L919X KI mutation, orthologous to the dominant R901X dRTA human mutation. This second mouse model exhibits the same inability to acidify urine at baseline as the R607H KI mouse line (Mumtaz et al., 2017), but overall has a milder phenotype. Additionally, after salt restriction in acid-loaded mice, we found that both L919X KI/KI and R607H KI/KI mice display persistent urinary sodium loss, as seen in patients with dRTA. The objectives of this work were to (1) characterize the L919X KI mouse line, (2) determine whether the two mouse models accurately reflect the urinary ion loss seen in patients with dRTA, and (3) begin investigating the mechanism causing urinary sodium loss in these *Ae1* mutant transgenic mice.

## RESULTS

### Generation of L919X KI mice and confirmation of decreased numbers of A-ICs

**Fig. 1.**
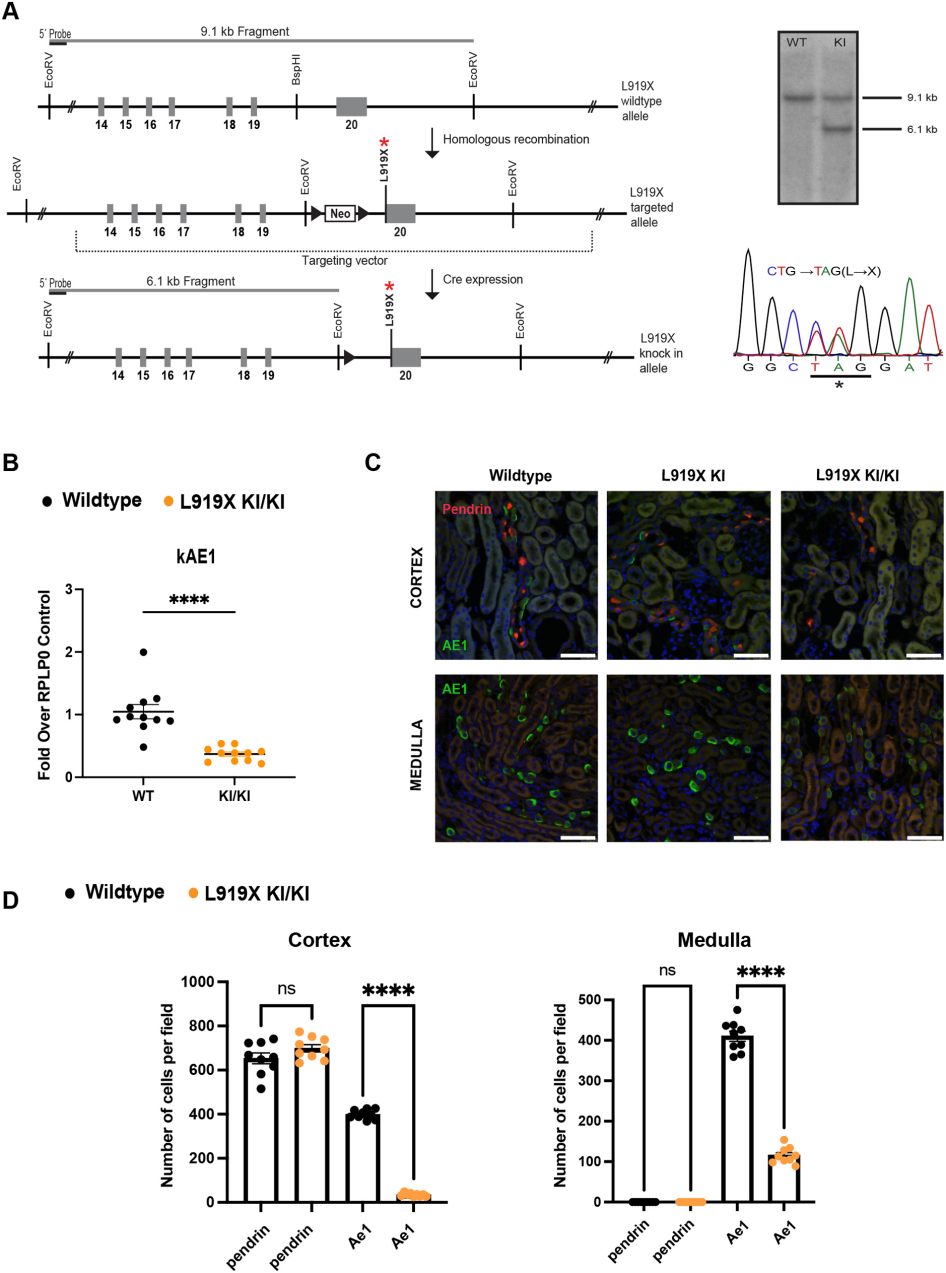
**Generation and characterization of the L919X KI mice.** (A) Left: targeting and screening strategy with murine *Ae1* locus (top), targeted locus (middle) and locus after removal of the selection cassette by Cre recombination (bottom). Right: Southern blotting results (top) and sequencing (bottom), validating the introduction of the mutation to the locus. KI, knock-in; WT, wild type. (B) Expression of *Ae1* in perfused whole kidney from WT or L919X KI/KI mice. (C) Immunofluorescence images from confocal microscopy of WT, heterozygous or homozygous L919X KI/KI mice medullary or cortical kidney sections stained with anti-pendrin (red) or anti-kAE1 (green) antibodies. Scale bars: 50 µm. (D) Quantification of type A (kAE1 positive) and type B (pendrin positive) intercalated cells in the cortex and medulla of WT or homozygous L919X KI mice. Error bars correspond to means±s.e.m. *n*=4-6 kidney sections. ns, not significant; ****P<0.0001 using unpaired two-tailed Student's *t*-test (B) or two-way ANOVA (D).

We generated mice carrying the orthologous mouse *Ae1* L919X mutation by homologous recombination (Fig. 1A). Homozygous mice were viable, developed normally and were fertile. Neither gross kidney malformations nor nephrocalcinosis were observed (Fig. S1A,B), similarly to the *Ae1* R607H mutant mice (DeFranco et al., 1995). Reverse transcription quantitative PCR (RT-qPCR) analysis of whole kidneys from WT and L919X KI/KI mice at steady state confirmed a significant decrease in *Ae1* mRNA expression in the mutant animals (Fig. 1B). Immunostaining for kAE1 in the cortex and medulla of WT, heterozygous and homozygous L919X KI mice (Fig. 1C,D) revealed that, similar to WT mice, the kAE1 protein predominantly localized to the basolateral membrane, indicating normal membrane targeting of the mutant protein with no evidence of apical localization (Fig. 1C). This finding is also consistent with previous findings in R607H KI mice (Mumtaz et al., 2017). Additionally, staining intensity for kAE1 in L919X KI/KI mice was markedly reduced compared to that in WT and heterozygous L919X KI mice, both in the cortex and the medulla. Conversely, signals for pendrin did not exhibit genotype-dependent differences (Fig. 1C,D). Collectively, these results suggest diminished abundance of kAE1-positive A-ICs in the L919X KI mouse model, consistent with R607H KI mouse observations.

### L919X KI/KI and R607H KI/KI mice excrete an alkaline urine coupled with less urinary ammonium than WT littermates

**Fig. 2.**
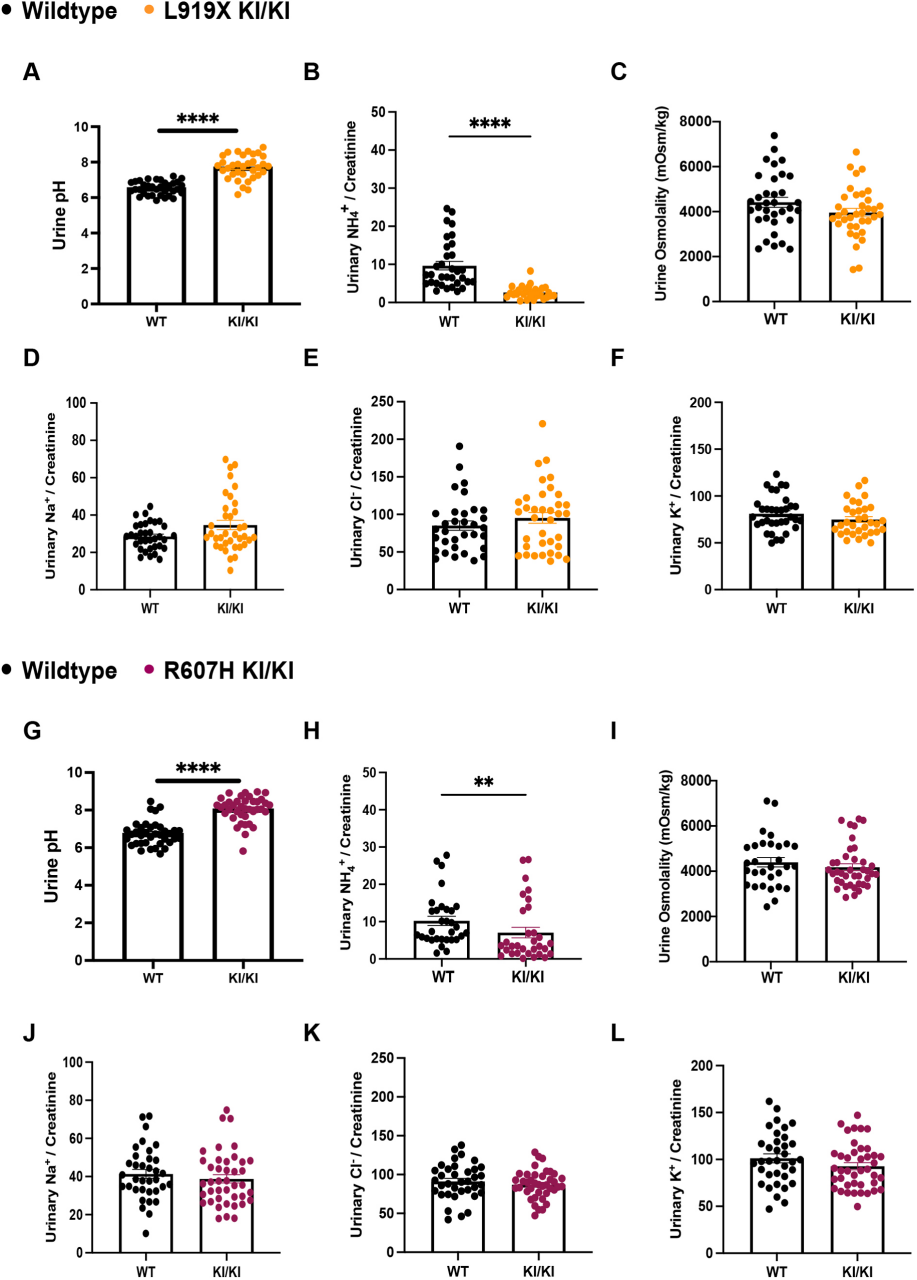
**At baseline, R607H KI/KI and L919X KI/KI mice exhibit alkaline urine and decreased urinary ammonium secretion.** (A-F) Urinary pH (A), ammonium/creatine ratio (B), osmolality (C), sodium/creatinine ratio (D), chloride/creatinine ratio (E) and potassium/creatinine ratio (F) comparison between WT and L919X KI/KI mice. (G-L) Urinary pH (G), ammonium/creatine ratio (H), osmolality (I), sodium/creatinine ratio (J), chloride/creatinine ratio (K) and potassium/creatinine ratio (L) comparison between WT and R607H KI/KI mice. Error bars correspond to means±s.e.m. **P<0.01, ****P<0.0001 using unpaired two-tailed Student's *t*-test or Mann–Whitney test. Note that the large sample size is due to the analysis of baseline urine samples from all experiments, resulting in 34-37 samples for WT and 35-39 samples for KI mice.

To validate the L919X KI/KI mice as a model of human dRTA, we assessed plasma and urine parameters at baseline (Fig. 2; Table S3). L919X KI/KI mice had alkaline urine compared to that of WT littermates (Fig. 2A). They also excreted significantly less urinary ammonium (Fig. 2B). Therefore, the L919X KI mice display the characteristic inability to properly acidify urine seen in patients with dRTA (Wagner et al., 2023). There was no difference in urine osmolality between genotypes (Fig. 2C), and the L919X KI/KI mice excreted sodium, chloride and potassium comparably to WT mice (Fig. 2D-F). There were no differences in plasma ion concentrations or pH between the L919X KI/KI and WT mice, although both mouse lines had low plasma bicarbonate and pH, likely due to anesthesia (Table S3). We completed a baseline analysis of the R607H KI/KI mice as well (Fig. 2G-L; Table S4), confirming that R607H KI/KI mice displayed the same phenotype as L919X KI/KI mice at baseline (Fig. 2G-L). These mutant mice had alkaline urine and decreased ammonium excretion (Fig. 2G,H), as previously shown (DeFranco et al., 1995). There was no difference in urine osmolality between the mutant and WT mice (Fig. 2I), and mutant mice also excreted sodium, chloride and potassium to a similar extent as WT mice (Fig. 2J-L). Overall, this baseline characterization supports an abnormal urinary acidification in both dRTA mouse lines at baseline, consistent with the hallmark characteristics of patients with dRTA (Mohebbi and Wagner, 2018).

### Acute sodium depletion confirms impaired urinary acidification and potassium excretion in dRTA mutant compared to WT mice

To assess whether dRTA mutant mice show a salt-losing phenotype similar to that of patients with dRTA, we challenged R607H KI/KI, L919X KI/KI mice or WT littermates with a sodium- and chloride-depleted diet for 24 h (Protocol 1) (Fig. S2, Tables S5 and S6). Both L919X KI/KI and R607H KI/KI mice consistently excreted urine with a higher pH than that excreted by WT littermates (Fig. S2A,F). Both mutant mice strains had no significant differences in urine osmolality between genotypes, although there was a trend towards lower osmolality for L919X KI/KI mice (Fig. S2B,G). Both L919X KI/KI and R607H KI/KI mice reduced urinary sodium and chloride excretion appropriately, to a similar extent as WT littermates (Fig. S2C,D,H,I). Interestingly, L919X KI/KI mice excreted less urinary potassium than that excreted by WT mice, while there was only a trend for reduced urinary potassium excretion in R607H KI/KI mice (Fig. S2E,J). No significant plasma difference was found between genotypes for each strain, except that R607H KI/KI mice continued to have increased plasma potassium compared to WT littermates (Tables S5 and S6). Together, these results provide preliminary evidence of renal electrolyte mishandling under acute sodium-depleted conditions for both L919X KI/KI and R607H KI/KI mice, suggesting defective coupling of potassium secretion to sodium and chloride reabsorption in the CDs of both strains.

### Salt-depleted acid loading results in hypernatremia and hyperchloremia, as well as increased renin levels, in both dRTA mutant strains

**Fig. 3.**
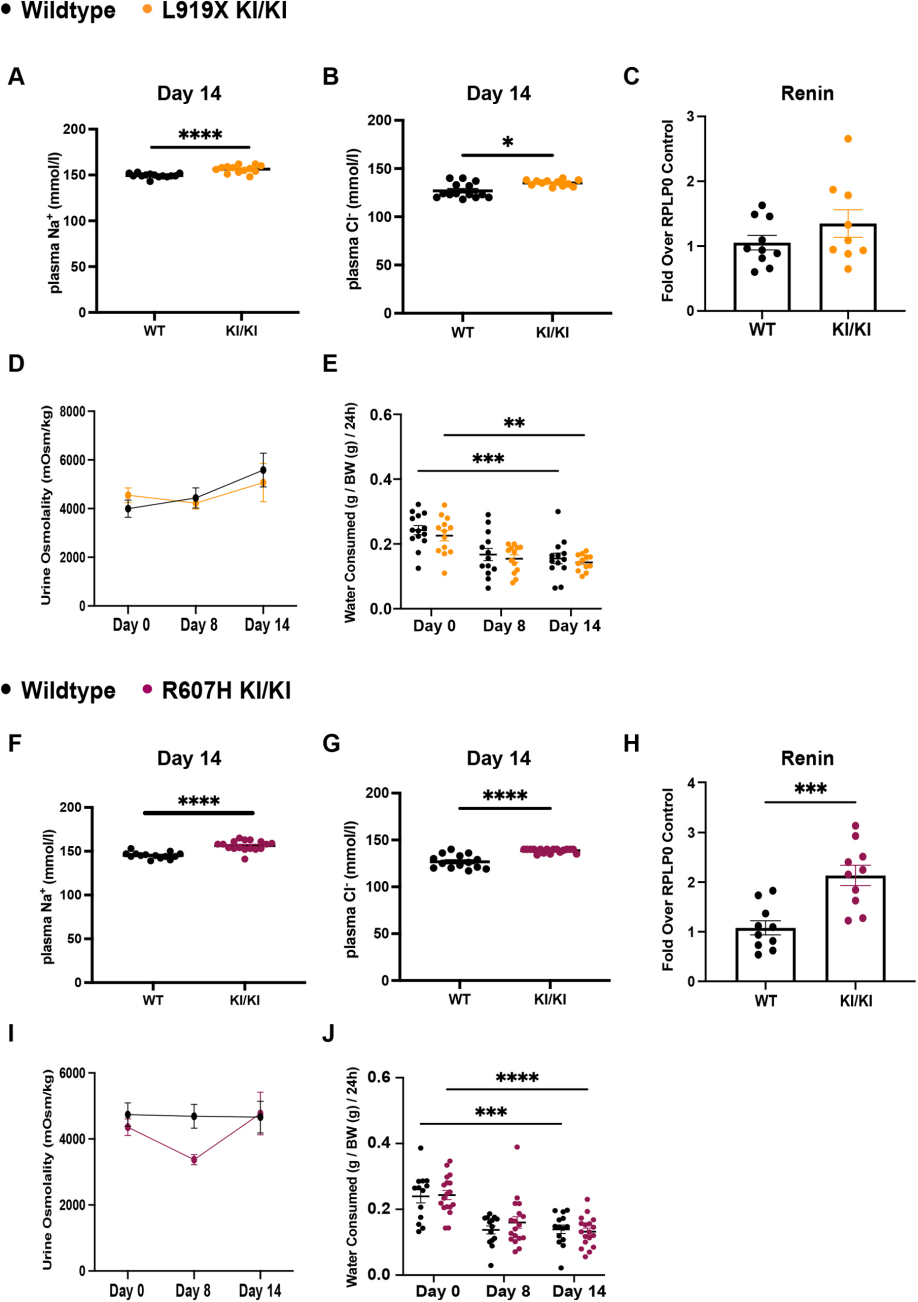
**After a salt-depleted acid diet, R607H KI/KI mice and L919X KI/KI mice are hypernatremic and hyperchloremic.** (A,B) Plasma sodium (A) and chloride (B) concentrations from L919X KI/KI mice or WT littermates. (C) Renin gene expression in L919X KI/KI mice or WT littermates. (D,E) Urine osmolality (D) and water consumed (E) by L919X KI/KI mice or WT littermates over the 14-day time course of the diet. BW, body weight. (F,G) Plasma sodium (F) and chloride (G) concentrations from R607H KI/KI mice or WT littermates. (H) Renin gene expression in R607H KI/KI mice or WT littermates. (I,J) Urine osmolality (I) and water consumed (J) by R607H KI/KI mice or WT littermates over the 14-day time course of the diet. Error bars correspond to means±s.e.m. *P<0.05, **P<0.01, ***P<0.001, ****P<0.0001 using unpaired two-tailed Student's *t*-test or two-way ANOVA with Tukey's multiple comparison test.

To further challenge the dRTA mutant mice, we fed WT and mutant mice a sodium-depleted acid diet (see Materials and Methods, Protocol 2). This diet was designed to recreate a study by Sebastian et al. (1976), revealing an urinary sodium-wasting phenotype in some patients with dRTA on a salt-restricted diet for 8-10 days (Sebastian et al., 1976). Following this challenge, only R607H KI/KI mice displayed metabolic acidosis from the acid load, with decreased plasma bicarbonate and pH (Table S8). L919X KI mice only showed a trend for acidosis (*P*=0.068) (Table S7), suggesting a more severe phenotype in R607H KI/KI mice than in L919X KI/KI mice. Notably, in both L919X KI/KI and R607H KI/KI mutant mice, plasma sodium and chloride concentrations were significantly increased compared to those in WT littermates and compared to steady state (Fig. 3A,B,F,G; Tables S7 and S8), suggesting a dehydrated state in KI mice. Given the significant hypernatremia and hyperchloremia in both mutant animals, we wondered whether the renin-angiotensin-aldosterone system (RAAS) or dehydration was activated in the mutant mice. Indeed, we observed higher renin mRNA expression in homozygous R607H KI mice than in WT mice, with a similar trend in L919X KI/KI mice (Fig. 3C,H). We next quantified water consumption (Fig. 3E,J), urine osmolality (Fig. 3D,I), and plasma hemoglobin and hematocrit levels for both strains (Tables S7 and S8). Although no difference in urine osmolality was observed compared to that in WT littermates (Fig. 3D,I), homozygous R607H and L919X mice had reduced water consumption (Fig. 3E,J), despite increased hemoglobin concentration in homozygous R607H mice (Table S8). These results suggest abnormal salt handling or impaired osmosensing, with activation of the RAAS and/or potential dehydration, in these mice.

### Salt-depleted acid loading reveals a salt-wasting nephropathy in homozygous L919X KI and R607H KI mice

**Fig. 4.**
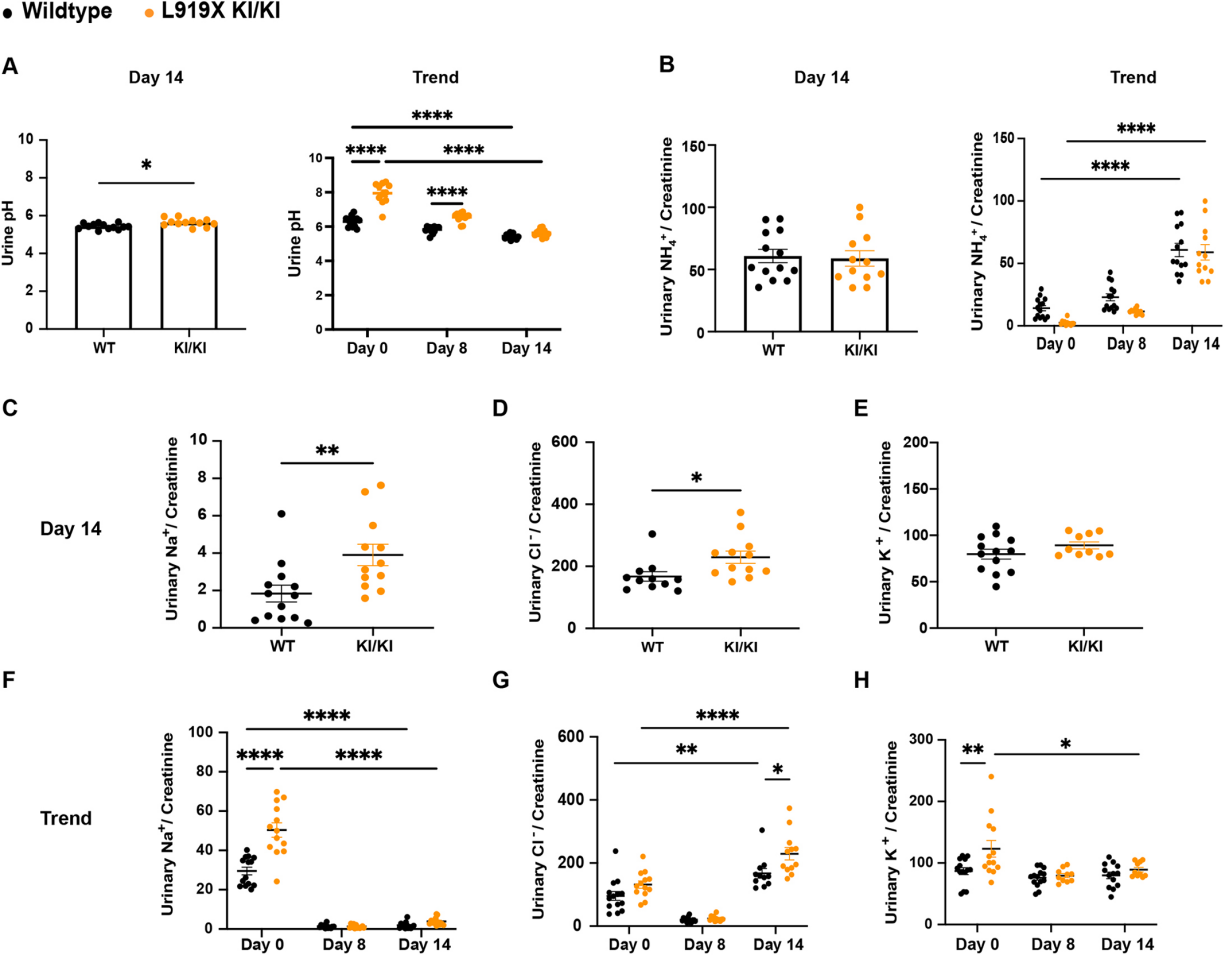
**After a salt-depleted acid diet, L919X KI/KI mice produce alkaline urine and waste urinary sodium and chloride.** (A) Urine pH on day 14 (left) and trend over the 14 experimental days (right) in L919X KI/KI mice or WT littermates. (B) Urinary ammonium/creatinine ratio on day 14 (left) and trend over the 14 experimental days (right) in L919X KI/KI mice or WT littermates. (C-E) Day 14 urinary sodium/creatinine ratio (C), sodium/chloride ratio (D) and potassium/creatinine ratio (E). (F-H) Trend in sodium/creatinine (F), sodium/chloride (G) and potassium/creatinine (H) ratios over the time course of the experiment in L919X KI/KI mice or WT littermates. Error bars correspond to means±s.e.m. *P<0.05, **P<0.01, ****P<0.0001 using unpaired two-tailed Student's *t*-test or two-way ANOVA with Tukey's multiple comparison test. Note that in F, no significant difference between WT and KI mice at Day 14 was detected, likely owing to the two-way ANOVA statistical test comparing multiple conditions and resulting in a high type I error and *P*-value, as a significant difference was found using an unpaired two-tailed Student's *t*-test (Fig. 3A).

**Fig. 5.**
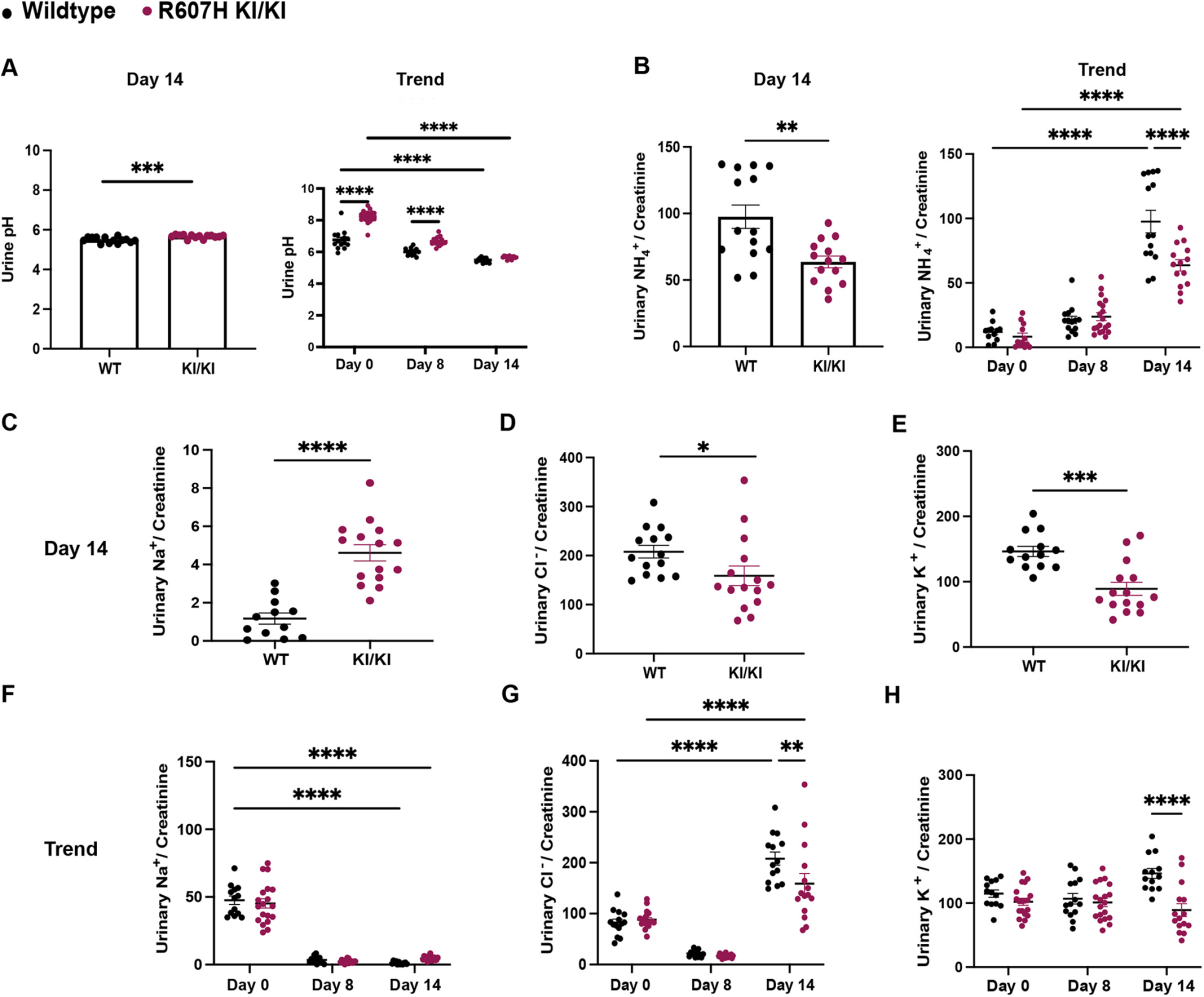
**After a salt-depleted acid diet, R607H KI/KI mice produce alkaline urine and waste urinary sodium and chloride.** (A) Urine pH on day 14 (left) and trend over the 14 experimental days (right) in R607H KI/KI mice or WT littermates. (B) Urinary ammonium/creatinine ratio on day 14 (left) and trend over the 14 experimental days (right) in R607H KI/KI mice or WT littermates. (C-E) Day 14 urinary sodium/creatinine ratio (C), sodium/chloride ratio (D) and potassium/creatinine ratio (E). (F-H) Trend in sodium/creatinine (F), sodium/chloride (G) and potassium/creatinine (H) ratios over the time course of the experiment in R607H KI/KI mice or WT littermates. Error bars correspond to means±s.e.m. *P<0.05, **P<0.01, ***P<0.001, ****P<0.0001 using unpaired two-tailed Student's *t*-test or two-way ANOVA with Tukey's multiple comparison test. Note that in F, no significant difference between WT and KI mice at Day 14 was detected, likely owing to the two-way ANOVA statistical test comparing multiple conditions and resulting in a high type I error and *P*-value, as a significant difference was found using an unpaired two-tailed Student's *t*-test (Fig. 3G).

Similarly to the sodium depletion diet alone, both homozygous L919X KI and R607H KI mice acidified their urine, although not to the same extent as their WT littermates (Fig. 4A and Fig. 5A). R607H KI/KI mice excreted significantly less ammonium than WT mice (Fig. 5B), whereas L919X KI/KI mice (Fig. 4B) showed no difference in ammonium excretion compared to their controls, although both genotypes had increased overall ammonium excretion at day 14 compared to day 0 (Fig. 4B and Fig. 5B). The salt-depleted acid diet also revealed a pronounced urinary sodium-losing phenotype in homozygous L919X KI and R607H KI mice (Fig. 4C,F and Fig. 5C,F), in agreement with observations from patients with dRTA under similar physiological conditions (Sebastian et al., 1976). Distinct phenotypes were observed concerning renal potassium and chloride handling (Fig. 4 and Fig. 5D,E,G,H). L919X KI/KI mice exhibited urinary chloride loss with no difference in potassium excretion compared to WT mice (Fig. 4D,E). In contrast, R607H KI/KI mice displayed lower urinary potassium and chloride excretion (Fig. 5D,E). These experiments demonstrate that, in mice, both the L919X and R607H mutation lead to urinary sodium loss and inability to maximally acidify their urine, thus reproducing major features observed in patients with type 1 dRTA.

### Paracellular proteins claudin 4 and claudin 10b are upregulated in dRTA mutant mice

**Fig. 6.**
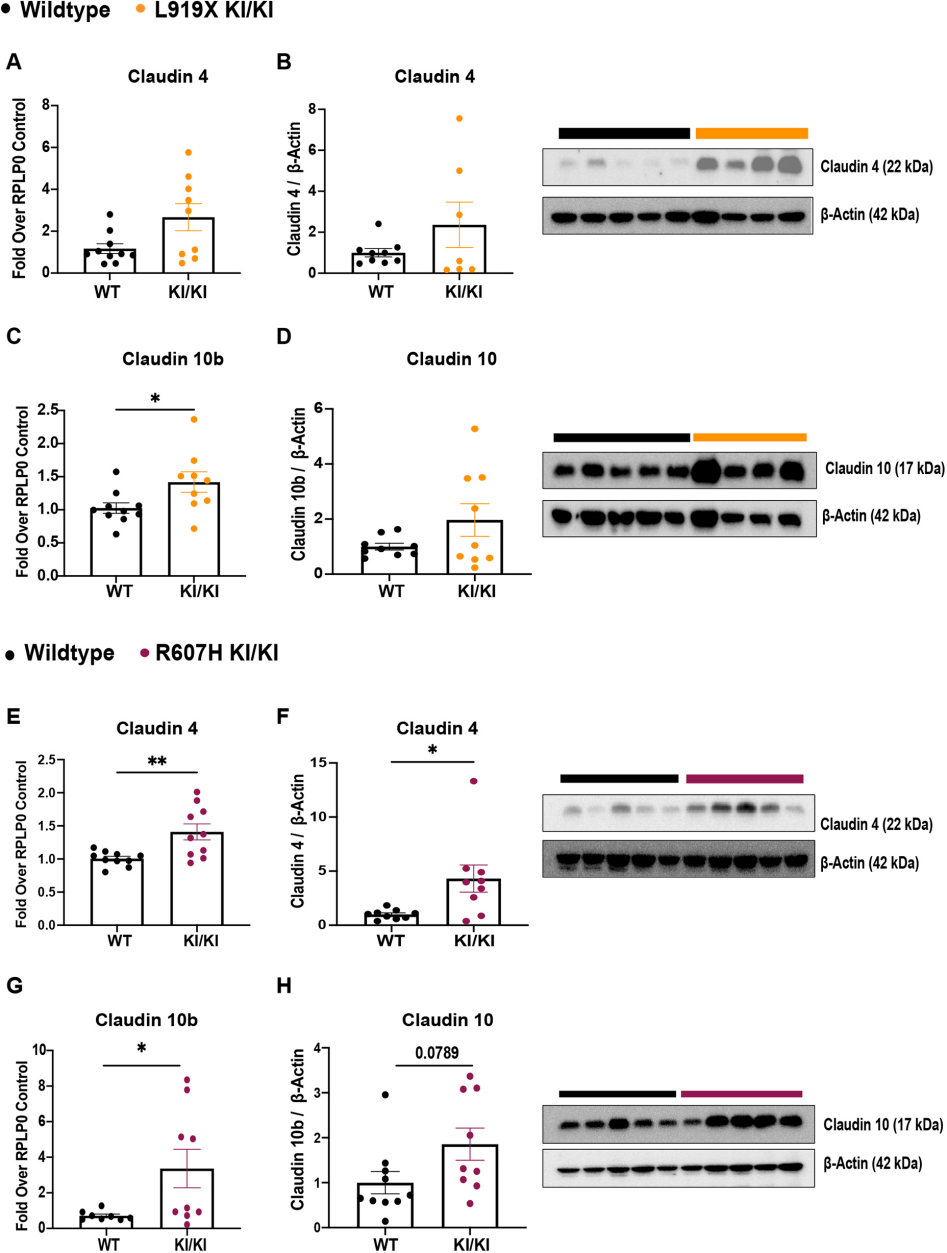
**Claudin 4 and claudin 10b are upregulated in L919X KI/KI mice and R607H KI/KI mice after a salt-depleted acid diet.** (A,B,E,F) Claudin 4 gene expression (A,E) and protein abundance (B,F), with a representative immunoblot image (right). Claudin 10b gene expression (C,G) and claudin 10 protein abundance (D,H), with a representative immunoblot image (right). Error bars correspond to means±s.e.m. *P<0.05, **P<0.01 using unpaired two-tailed Student's *t*-test or two-way ANOVA with Tukey's multiple comparison test.

Given the alkaline urine and urinary sodium loss after salt-depleted acid loading, we quantified mRNA and protein levels of specific markers of the CD, the thick ascending limb (TAL) and the distal convoluted tubule (DCT) in KI/KI mice and their respective WT littermates (Fig. 6; Figs S3 and S4).

We found increased mRNA and protein levels of claudin 4 and claudin 10b, in both L919X KI/KI and R607H KI/KI mice (Fig. 6A-H). Claudin 4 is a paracellular chloride pore and a sodium pore blocker expressed in the thin ascending limb of the loop of Henle, DCT and CD (Chambrey and Trepiccione, 2015; Hou et al., 2013; Leiz and Schmidt-Ott, 2020), whereas claudin 10b is a paracellular sodium pore in the TAL (Yu, 2015; Quintanova et al., 2022; Hou et al., 2010). These findings suggest a common compensatory mechanism via the paracellular pathway in the loop of Henle of both mutant strains.

The salt-depleted acid challenge also revealed downregulation of A-IC and B-IC marker gene expression in R607H KI/KI mice, with similar trends in L919X KI/KI mice (Fig. S3 and Fig. S4A-I). There was no clear trend for principal cell markers between KI/KI mice and WT littermates. In agreement with decreased numbers of ICs reported for R607H KI/KI mice (Mumtaz et al., 2017), kAE1 and B1-ATPase protein or mRNA levels in whole kidney lysate were significantly reduced compared to those in WT littermates (Fig. S3 and Fig. S4A,C,K). Pendrin and *Ae4* (also known as *Slc4a9*) mRNA levels were significantly reduced in R607H KI/KI mice compared to those in WT mice, with a similar trend in L919X KI/KI mice (Fig. S3 and Fig. S4C,E). Increased *Ndcbe* mRNA expression was observed in R607H KI/KI mice compared to that in WT mice, with a similar trend in L919X KI/KI mice (Fig. S3 and Fig. S4D).

Given the higher abundance of the TAL-specific claudin 10b in both mutant mouse lines (Fig. 6C,D,G,H), we investigated gene expression of various transporters from prior nephron segments (Fig. S3 and Fig. S4H-J,M). mRNA expression of the DCT sodium-chloride cotransporter (*Ncc*; also known as *Slc12a3*) was significantly lower in L919X KI/KI mice than in WT mice, with a similar trend in R607H KI mice (Fig. S3 and Fig. S4J). We finally assessed the mRNA expression of the sodium/potassium/chloride cotransporter *Nkcc2* (also known as *Slc12a1*) and sodium/proton exchanger *Nhe3* (also known as *Slc9a3*) in whole kidneys, with the caveat that NHE3 is also abundantly expressed in the proximal tubule. These levels did not differ between L919X KI/KI and WT mice, but were significantly decreased in R607H KI/KI mice compared to WT littermates (Fig. S3 and Fig. S4H,I). Overall, these results highlight paracellular adaptations in both KI/KI mice, potentially in an attempt to maintain salt homeostasis.

## DISCUSSION

In this study, we characterized a second dRTA mouse model that carries the murine *Ae1* L919X mutation orthologous to the human dominant R901X dRTA mutation. We focused on homozygous mice as they display a stronger phenotype than heterozygotes, which is more representative of the symptoms of patients with dRTA. In agreement with the previously published R607H KI/KI model, at baseline, the L919X KI/KI mice exhibit an alkaline urine compared to that of WT littermates, mimicking the main physiological defect observed in patients with dRTA. In perfused kidneys from homozygous L919X and R607H KI mice, we observed decreased *Ae1* gene expression, suggesting loss of A-ICs. Overall, our results support that both L919X KI/KI and R607H KI/KI mouse lines reproduce the alkaline urine seen in patients with dRTA and thus are good models to investigate the underlying pathophysiology.

Our results also reveal a more pronounced phenotype in R607H KI/KI compared to L919X KI/KI mice, with slight differences in mRNA levels of markers of the loop of Henle and CD. One study reporting dominant dRTA mutations in the *SLC4A1* gene found that, despite phenotypic similarities, the R901X mutation caused a milder phenotype than the R598H mutation, including lower plasma bicarbonate concentration and more severe hypokalemia, in patients with the R589H mutation than in those with the R901X mutation (Karet et al., 1998). Our results confirm this trend, with significant differences observed in R607H KI/KI mice versus WT littermates but only similar trends in the L919X KI mice.

We challenged homozygous *Ae1* L919X KI/KI and R607H KI/KI mice and their respective controls with an acute salt-depleted diet or a salt-depleted diet in combination with an acid challenge to assess whether these mice develop a sodium-wasting phenotype as shown for some patients with dRTA (Sebastian et al., 1976). Both dRTA mutant mice exhibited renal sodium loss compared to WT controls. Both KI/KI mice had elevated plasma sodium and chloride levels, with increased abundance of renal renin mRNA, suggestive of a volume-contracted state despite a similar volume of water consumed and urine osmolality. Importantly, we found an increase in claudin 4 and claudin 10b gene and protein expression in homozygous L919X KI and R607H KI mice compared to WT mice. A previous study feeding a salt-restricted diet to a B1-H^+^-ATPase knockout mouse model elucidated one mechanism: loss of *Atp6v1b1* impaired sodium reabsorption via pendrin/NDCBE in B-ICs (Gueutin et al., 2013). These mice also had increased urinary ATP and prostaglandin E2 (PGE2) release (known sodium movement inhibitors), which decreased ENaC expression in PCs, further contributing to sodium wasting (Gueutin et al., 2013). Consistent with these findings, our experiments showed decreased pendrin and *Ae4* gene expression levels, but increased *Ndcbe* gene expression levels, in R607H KI/KI mice. We also observed increased γ-ENaC (*Scnn1g*) mRNA and protein expression in R607H KI/KI mice compared to WT mice, but no significant difference in renal outer medullary potassium channel *Romk* (also known as *Kcnj1*) gene expression. These trends were also observed in L919X KI/KI mice, with the additional finding of decreased *Ncc* mRNA expression.

Both mouse lines displayed similar alterations in their paracellular pathway, as an increase in claudin 4 (Giglio et al., 2021; Fry and Karet, 2007; Hou et al., 2010) and claudin 10b (Leiz and Schmidt-Ott, 2020) mRNA expression was observed after acid challenge with salt depletion. Claudin 4 functionally interacts with kAE1, as expressing the exchanger in immortalized inner medullary collecting duct cells increases transepithelial permeability to both sodium and chloride in a mechanism dependent, in part, on claudin 4 and with-no-lysine kinase 4 (Lashhab et al., 2023; 2019b). Although present in tight junctions, claudin 10 also colocalizes with the sodium/potassium ATPase and chloride channels in the basolateral infoldings of the TAL (Quintanova et al., 2022). Although its role there remains unclear, it could play a crucial role in facilitating high electrolyte flow and preventing basolateral water influx. Therefore, increased expression of claudin 10b could represent a compensatory mechanism to enhance urine concentration and regulate electrolyte balance, and provides evidence that disruptions in transcellular mechanisms impact paracellular pathways in the nephron.

The upregulation of claudins including in the loop of Henle points towards a possible compensatory role of this segment in urinary acidification and sodium reabsorption. The ascending limb of the loop of Henle is a site for urinary acidification via the combined action of apical NHE3 and basolateral NBCn1 (also known as SLC4A7) (de Bruijn et al., 2015; Olsen et al., 2021). As shown by our experiments, mutant mice still acidify their urine despite being kAE1 depleted, which points towards a possible contribution of this segment to urinary acidification, in addition to sodium and chloride reabsorption. The function of acid-base transporters is tightly coupled to that of salts in the TAL, through apical NHE3 and the basolateral electroneutral sodium bicarbonate co-transporter, NBCn1 (de Bruijn et al., 2015; Olsen et al., 2021). NBCn1 facilitates urine acidification during metabolic acidosis (Olsen et al., 2021), promoting ammonium reabsorption across the renal tubules, rapidly restoring arterial blood pH and facilitating its secretion by the CD (Ares et al., 2011). Previous work reported that R607H KI/KI mice have increased NCBn1 protein abundance at steady state compared to WT mice (DeFranco et al., 1995). The potential upregulation of this protein in the KI/KI mice lines could provide an explanation for their ability to effectively regulate plasma pH and acidify their urine to some extent despite the loss of ICs. Additionally, NKCC2 and ROMK in the TAL might cause a net chloride reabsorption, creating a lumen-positive transepithelial potential driving paracellular sodium reabsorption through claudin 10b.

After the salt-depleted acid challenge, we detected significantly decreased *Nhe3* and *Nkcc2* mRNA expression in homozygous R607H KI mice compared to WT mice. The relevance of this finding may be limited as both NHE3 and NKCC2 proteins are post-translationally regulated. NKCC2 regulation occurs via membrane trafficking, phosphorylation and protein-protein interactions (Ares et al., 2011). NHE3 is regulated through phosphorylation, and its activity can be inhibited by aldosterone (Girardi and di Sole, 2012; Watts et al., 2006), which may be relevant in the context of salt depletion.

There are some limitations to this work. Although the KI/KI mice reproduced some symptoms of dRTA, they are not exhibiting them all. First, these mice knocked in with a dominant dRTA mutation display a milder phenotype than that of patients with dRTA. Indeed homozygous mice exhibited acidemia only after an acid challenge (Mumtaz et al., 2017), whereas heterozygous patients with dRTA with dominant mutations have overt acidemia at baseline. We hypothesize that this is due to the differences in diet between rodents and humans. Therefore, we focused our study on homozygous mice. Second, these mice do not exhibit hypercalciuria, nephrocalcinosis and kidney stones, which are commonly observed in patients with dRTA. The reason for this discrepancy is unknown. Importantly, mice with a disruption on *Atp6v1b1*, the ortholog of which in humans causes dRTA, also lack the hypercalciuria, nephrocalcinosis and overt acidemia (Gueutin et al., 2013) seen in these patients, pointing to some limitations inherent to these mouse models. However, although a limitation, the lack of these symptoms allowed us to investigate other dRTA symptoms without the caveats of kidney stones and nephrocalcinosis.

Overall, our results show that both L919X KI/KI and R607H KI/KI mice recapitulate some dRTA symptoms, including the urinary sodium loss seen in patients. Our results reveal physiological mechanisms for this complex disease and point to potential compensatory acidification and salt reabsorption in earlier nephron segments.

## MATERIALS AND METHODS

### Generation of the L919X KI mice

In Germany, animal experiments were approved by the Thüringer Landesamt für Verbraucherschutz (TLV) under the license 02-016/14. In Canada, animal protocols were approved by the University of Alberta's Animal Care and Use Committee (AUP #1277) and were in accordance with national and institutional animal care guidelines. At baseline, mice were maintained on a 12-h light/dark cycle with drinking water and standard rodent chow (PicoLab Mouse Diet 20 #5058) available *ad libitum*. R607H KI mice (129/SvJ strain) have previously been published, and their generation has been described (Mumtaz et al., 2017).

L919X KI mice were generated by homologous recombination similar to *Ae1* R607H KI mice (DeFranco et al., 1995). Briefly, a 14.7 kb genomic DNA fragment (GRCm38/mm10; chr11:102,341,468-102,356,137) from a lambda phage library including exons 14-20 of the *Slc4a1* gene was cloned into the pKO-DTA Scrambler vector (Lexicon Genetics) containing a phosphoglycerate kinase (Pgk) promoter-driven diphteria toxin A cassette. A neomycin resistance cassette flanked by loxP sites and an 5′ EcoRV site were introduced at the BspHI restriction site in the genomic *Ae1* sequence. Site-directed mutagenesis was used to generate the L919X mutation in the adjacent exon 20. The linearized targeting vector was electroporated into R1 mouse embryonic stem cells. Clones resistant to Neomycin were analyzed by Southern blotting on EcoRV-digested genomic DNA with an external probe (GRCm38/mm10; chr11:102,356,140-102,356,838) labeling a 9.1 kb WT or 6.1 kb KI fragment (Fig. 1A). Correctly targeted embryonic stem cell clones were injected into C57BL/6 blastocysts to obtain chimeric mice. Chimeric mice with germ line transfer were bred with a Cre-Deleter mouse strain to excise the Neomycin resistance cassette. Heterozygous KI mice were backcrossed for at least five generations with C57BL/6 mice before WT and homozygous KI (KI/KI) mice were bred for experiments. Mice were genotyped by PCR of ear notch biopsies using primers for the R607H mice (5′-TAGCTCCTTCTACCCCACCCA-3′ and 5′-CCAGAGGTACATGGTAAAACATTGTC-3′), as previously published (Mumtaz et al., 2017), and primers for the L919X mice (5′-GCTTCCGTCTTGGTCTGCTGTG-3′ and 5′-TGGACAAGCCCTGCTGTCCCTA-3′), detecting a 301 bp band for WT allele and a 444 bp for the KI allele.

### Metabolic cage experimental design and diets

For each diet, 2- to 4-month-old homozygous KI (R607H or L919X) mice and WT littermates (equal number of male and female mice) were placed in metabolic cages (Tecniplast) for 72 h to acclimate and fed a standard rodent chow and water (PicoLab Mouse Diet 20 #5053, containing 0.3% sodium and 0.53% chloride), available *ad libitum*. Following this period, mice were fed one of two diets: Protocol 1 (‘salt depletion’) or Protocol 2 (‘salt depletion with acid load’). For Protocol 1, R607H KI/KI mice, L919X KI/KI mice and WT littermates were fed a salt-restricted diet (0.01-0.02% sodium, 0.07% chloride, Teklad Custom Diet #TD.08290) and given regular water *ad libitum* for 24 h. For Protocol 2, R607H KI/KI mice, L919X KI/KI mice and WT littermates were fed a low-salt diet for 8 days (Teklad Custom Diet #TD.08290). On day 9, the mice continued this salt restriction but were provided with acid in their drinking water (0.28 M NH_4_Cl with 0.5% sucrose), available *ad libitum* for 6 additional days. Fresh acid water was replaced every day. The total experimental period was 14 days.

Body weight, chow and water consumption, urine (under mineral oil) and feces measurements were collected every 24 h. On the final experimental day of Protocol 1 and 2, mice were anesthetized with a 50 mg/kg dose of pentobarbital. Blood was collected in lithium heparin-coated tubes and centrifuged for 10 min at 18,500 ***g*** at 4°C to collect serum, which was then stored at −80°C. After PBS/heparin perfusion, whole kidneys were dissected into four halves, and each half was processed for RNA extraction, immunoblotting, immunofluorescence or immunohistochemistry, and then stored at −80°C.

### Urine and serum analysis

Freshly retro-orbitally collected blood was analyzed for electrolytes (Na^+^, K^+^ and Cl^−^), glucose, urea nitrogen, hematocrit, hemoglobin and pH using an i-STAT1 Analyzer (Abaxis) with an i-STAT Chem8+ cartridge chip (Abbott Laboratories). Urine pH was measured using a pH microelectrode (PerpHect Ross Micro Combination pH electrode, Thermo Fisher Scientific) attached to a Accumet AR10 pH meter (Fisher Scientific). Urine osmolality was measured with the Advanced Instruments Osmo1 Single-Sample Micro-Osmometer (Thermo Fisher Scientific), diluting samples at 1:10 or 1:100 with filtered ddH_2_O. Urine electrolytes were measured by ion chromatography (Dionex Aquion Ion Chromatography System, Thermo Fisher Scientific) with an autosampler. Urine samples were diluted 1:100 or 1:250 with ddH_2_O, and a 4.5 mM Na_2_CO_3_/1.5 mM NaHCO_3_ in ddH_2_O solution was used for anion eluent and a 20 mM methanesulfonic acid in ddH_2_O solution for cation eluent composition (Plain et al., 2020). Urine creatinine was measured using a parameter creatinine kit (R&D Systems) or with ion chromatography. Chromeleon 7 Chromatography Data System software (Thermo Fisher Scientific) was used to analyze results. Urine ions were normalized to urinary creatinine concentration.

### RNA isolation and RT-qPCR

Immediately after collection, a half kidney was incubated in RNAlater (Thermo Fisher Scientific) for 8 h at room temperature before storing at −80°C. Total RNA was extracted using TRIzol reagent (Invitrogen) as previously described (Grinstein et al., 2018). RNA quantification was performed using a NanoDrop spectrophotometer (NanoDrop 2000C, Thermo Fisher Scientific). Then, 2 µg of cDNA was reverse transcribed with SuperScript™ II (Invitrogen) as per the manufacturer's instructions. The cDNA was pooled to create a serially diluted standard curve. Real-time quantitative PCR was performed in triplicate for each cDNA sample using TaqMan PCR master mix in a QuantStudio 6 Pro RT PCR system (Thermo Fisher Scientific). Samples were quantified using the 2^–ΔΔCt^ method (Livak and Schmittgen, 2001). Expression levels of mRNA from specific genes were normalized to housekeeping ribosomal protein lateral stalk subunit P0 (*Rplp0*) gene. Primers for the genes of interest are listed in Table S1.

### Protein extraction and immunoblot

A quarter of a freshly dissected kidney was decapsulated and mechanically homogenized in ice-cold lysis buffer (0.3 M sucrose, 25 mM imidazole, 1 mM EDTA, 8.5 µM leupeptin, 1 mM phenylmethylsulfonyl fluoride) (Kim et al., 2012), vortexed every 15 min over 1 h, and centrifuged at 4°C, 18,500 ***g*** for 15 min. Protein concentration was measured using a Bicinchoninic Acid Protein Assay (Pierce). Immunoblot experiments were performed with 8%, 10% or 12% SDS-PAGE gels, transferred to a polyvinylidene fluoride membrane and blocked with 3% milk in TBST (5 mM Tris base, 15 mM NaCl, 0.1% Tween 20). Membranes were incubated in primary antibodies overnight at 4°C followed by incubation in secondary antibodies conjugated to horseradish peroxidase. The antibodies used are listed in Table S2. Proteins were visualized using a Clarity Western ECL kit (Bio-Rad), and images were captured by a ChemiDoc touch imaging system (Bio-Rad). Quantification and densitometric analysis were performed by ImageJ (National Institutes of Health).

### Immunostaining

Freshly collected kidneys retrogradely perfused with paraformaldehyde (4%) were flash frozen in liquid nitrogen-cooled isopentane. Cryosections (4 µm thickness) were prepared and subjected to Masson-Goldner or Von Kossa staining, or further processed for immunostaining as previously described (López-Cayuqueo et al., 2018).

### Statistical analysis

Statistical analysis was completed using GraphPad Prism software (Ver 7.0e). Normality was verified for all datasets, and outliers (as determined by Prism) were removed. Analysis was performed using unpaired two-tailed Student's *t*-test or Mann–Whitney test, or two-way ANOVA where appropriate. *P*<0.05 was considered statistically significant. All data are presented as mean±s.e.m.

## Supplementary Material

10.1242/dmm.052138_sup1 Supplementary information
