# Supplementary material for: Urinary sodium wasting and disrupted collecting duct function in mice with distal renal tubular acidosis mutations
Source: Dis Model Mech. 2025 May 23;18(5):dmm052138. doi: 10.1242/dmm.052138 (PMC12128221; doi:10.1242/dmm.052138)
Supplement: Supplementary information [file dmm-18-052138-s1.pdf]

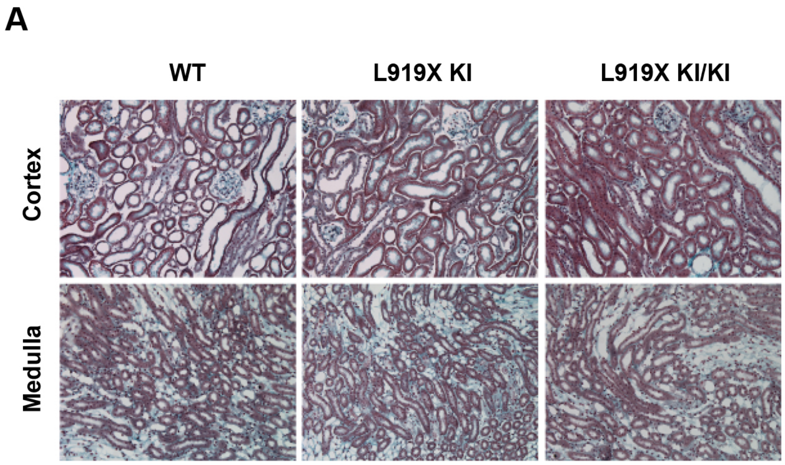

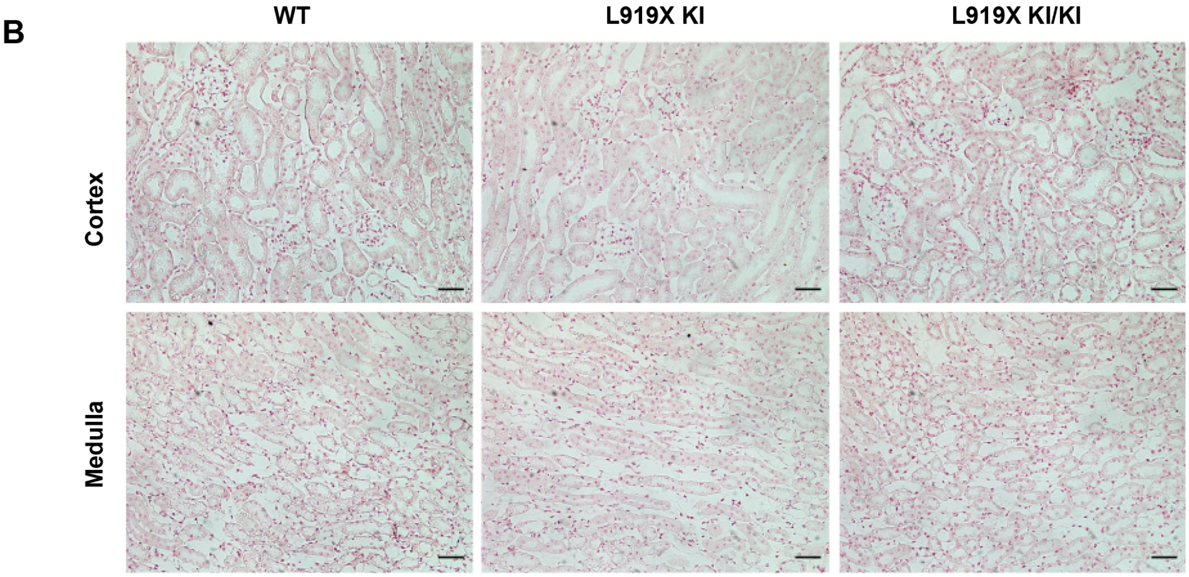

Fig. S1. Normal kidney structure and absence of nephrocalcinosis in Ae1 L919X KI mice. Masson Trichrome (A) and Von Kossa (B) staining of WT, heterozygous (L919X KI) or homozygous (L919X KI/KI) mouse cortical or inner stripe of outer medullary kidney sections.

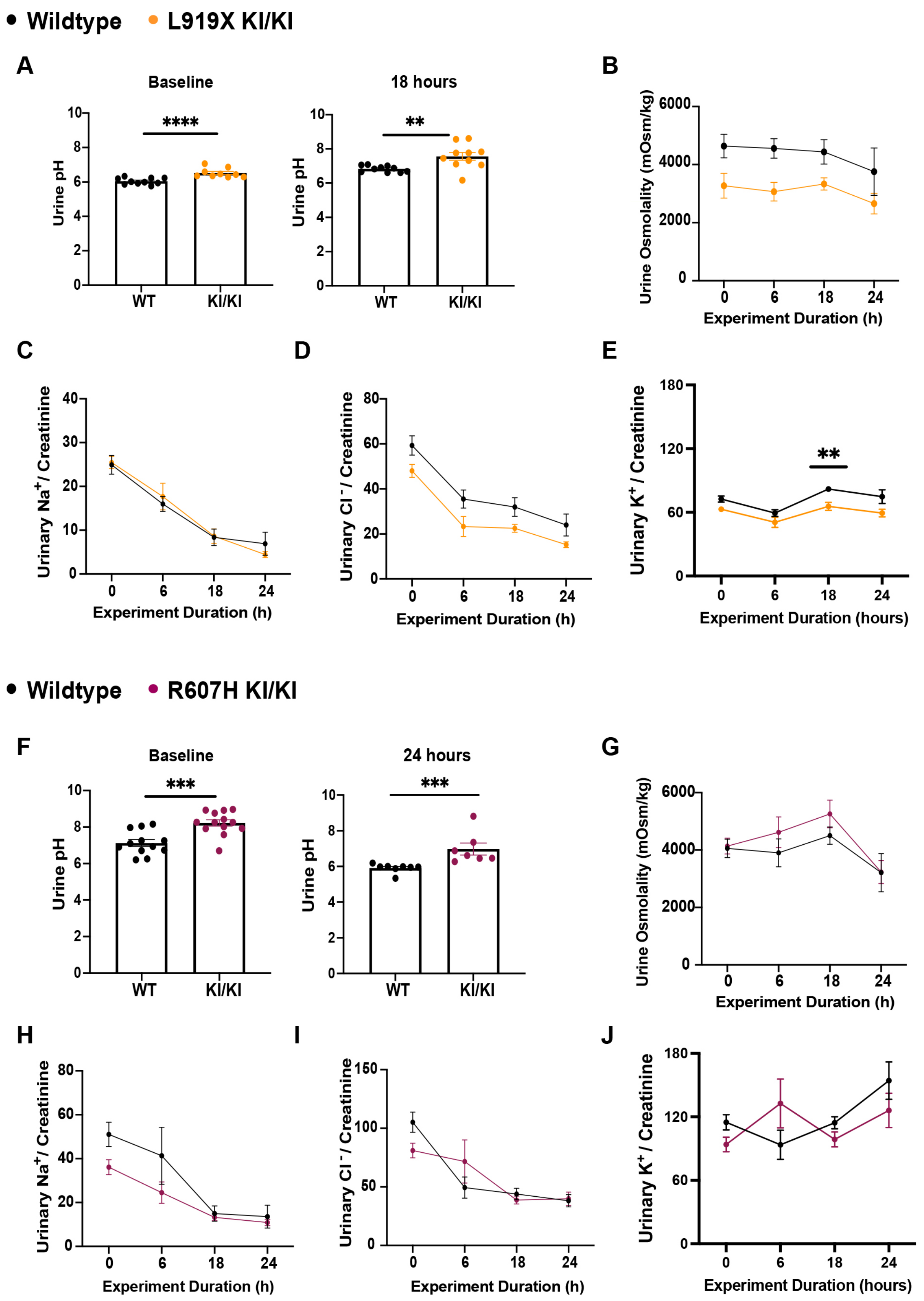
Wildtype
L919X KI/KI
A
Baseline
****
Urine pH
WT
KI/KI
18 hours
**
B
Urine Osmolality (mOsm/kg)
Experiment Duration (h)
C
Urinary Na+/ Creatinine
D
Urinary Cl-/ Creatinine
E
Urinary K+/ Creatinine
Experiment Duration (hours)
R607H KI/KI
F
***
24 hours
G
H
I
J

Fig. S2. After a 24h salt-depleted diet, both R607H and L919X KI/KI mice continue to produce an alkaline urine compared to WT littermates. (**A**) urinary pH at baseline (left) and after 18 h of diet (right) in WT or Ae1 L919X KI/KI mice. Urine osmolality (**B**), urinary sodium/creatinine ratio (**C**), urinary chloride/creatinine ratio (**D**) and urinary potassium/creatinine ratio (**E**) over the course of the 24h experiment in WT or L919X KI/KI mice. (**F**) urinary pH at baseline (left) and after 18 h of diet (right) in WT or Ae1 R607H KI/KI mice. Urine osmolality (**G**), urinary sodium/creatinine ratio (**H**), urinary chloride/creatinine ratio (**I**) and urinary potassium/creatinine ratio (**J**) over the course of the 24h experiment in WT or L919X KI/KI mice. Error bars correspond to means ± SEM, **P < 0.01, ***P < 0.001, ****P < 0.0001 using Student's t-test or Mann-Whitney test, or two-way ANOVA with Tukey's multiple comparison test.

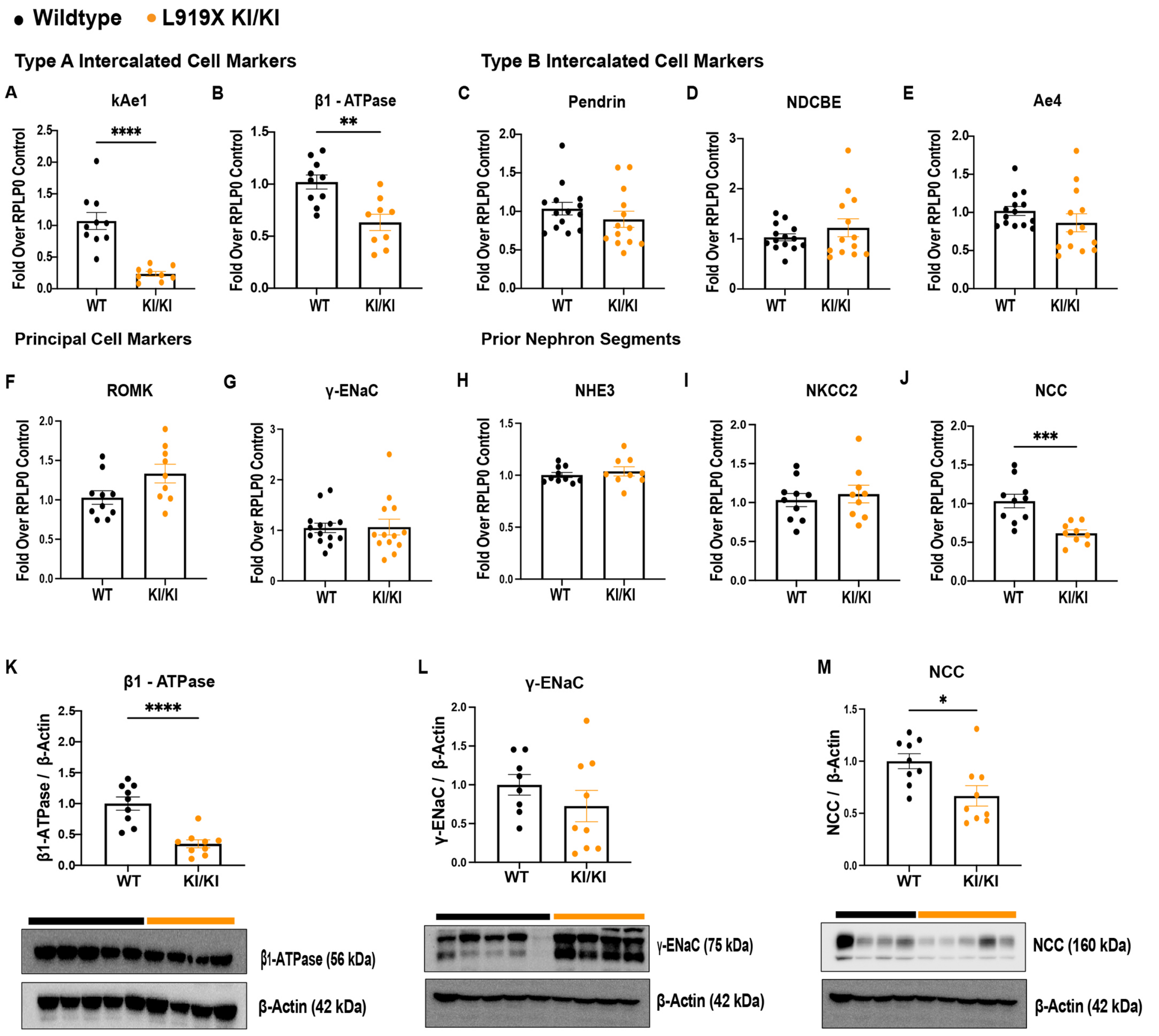

Fig. S3. Comparison of gene expression and protein abundance of markers of the IC, PC and other nephron segments in L919X KI/KI mice or WT littermates. Gene expression of A-IC markers kAe1 (**A**), B1-ATPase (**B**), B-IC markers pendrin (**C**), NDCBE (**D**), and Ae4 (**E**), PC markers ROMK (**F**) and gamma-ENaC (**G**), other markers NHE3 (**H**), NKCC2 (**I**) and NCC (**J**). Relative protein abundance of B1-ATPase (**K**), gamma-ENaC (**L**) and NCC (**M**) with representative immunoblot images (below). Note that the same membrane was used to detect gamma-ENaC and NCC, so the same image for beta-actin is shown and was used for quantification. Error bars correspond to means ± SEM, *P < 0.05, **P < 0.01, ***P < 0.001, ****P < 0.0001 using Student's t-test.

Disease Models & Mechanisms • Supplementary information

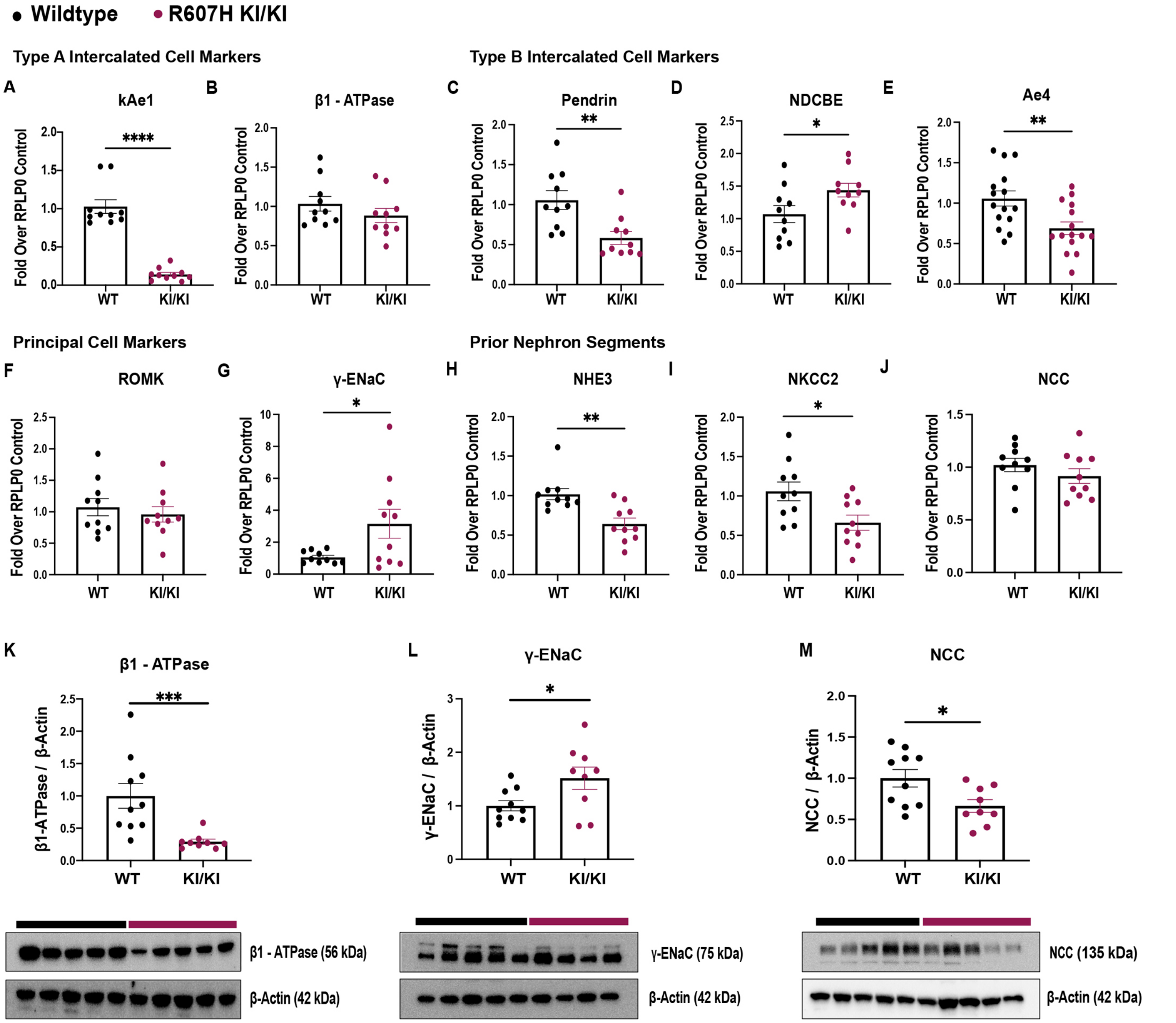

Fig. S4. Comparison of gene expression and protein abundance of markers of the IC, PC and other nephron segments in R607H KI/KI mice or WT littermates. Gene expression of A-IC markers kAe1 (**A**), B1-ATPase (**B**), B-IC markers pendrin (**C**), NDCBE (**D**), and Ae4 (**E**), PC markers ROMK (**F**) and gamma-ENaC (**G**), other markers NHE3 (**H**), NKCC2 (**I**) and NCC (**J**). Relative protein abundance of B1- ATPase (**K**), gamma-ENaC (**L**) and NCC (**M**) with representative immunoblot images (below). Error bars correspond to means ± SEM, *P < 0.05, **P < 0.01, ***P < 0.001, ****P < 0.0001 using Student's t-test.

Disease Models & Mechanisms • Supplementary information

Table S1. Primers and probes used for qPCR on mouse genes.

Name	Forward	Reverse	Probe
AE4<br>NM_N172830	5'-<br>CTCTGTGTCTTTGAGCTTTGC-<br>3'	5'-<br>TCTGCCTATGGTTGCTT<br>CTG-3'	5'-/56-<br>FAM/TGTTTCCTC/ZEN/CTGTGCC<br>TGGGTATTG/3IABKFQ/-3'
alpha-ENaC<br>NM_011324.2	5'-<br>CAGTGATGTCCCTGTCAAGAA<br>-3'	5'-<br>CTTGGGCTTAGGGTAGA<br>AGATG-3'	5'- /56-FAM/TGA TCA AGA<br>/ZEN/AGT GTGGCT GTG CCT<br>/3IABKFQ/ -3'
Atp6v1b1<br>NM_134157.1	5'-<br>CTTCTCACACTGGTAGGCAAG<br>-	5'-<br>AGTCAGATTTCGAGCAG<br>AATGG-3'	5'-/56-<br>FAM/CCGCTCAAT/ZEN/CGTAGGG<br>TCATTGGC/3IABkFQ/-3'
Cldn10b<br>NM_N021386.1	5'-<br>GCAGCGATCATTAGTCCTCTA<br>C-3'	5'-<br>CCACAGCCACTTATTTT<br>GCC-3'	5-/56-FAM/A ATCGGTA<br>A/ZEN/CGCAGATCTCCACAGG/3IA<br>BkFQ/-3'
Cld4<br>NM_N009903.1	5-<br>'CAGGACTGCCAAGGAGATTC-<br>3'	5-<br>'AACACTTTCTCAGCCCT<br>CTG-3'	5-'/56-<br>FAM/CATAGACGC/ZEN/CATCGCT<br>CAGCCTC/3IABkFQ/-3*
gamma-ENaC<br>NM_N011326.1	5'-<br>GTCAGAGGTGTCATTTGAGCA<br>-3'	5'-<br>GAGAACGAGAAGGGAA<br>AGGC-3'	5'-/56-<br>FAM/TCGGAAGCG/ZEN/GAAAATC<br>AGGGGAA/3IABKFQ/-3'
kAE1<br>NM_009902.4	5'-<br>AAGAGGTCAAGGAACAGCG-3'	5'-<br>GTACAGGAAGATGCCG<br>AAGAG-3'	5-/56-<br>FAM/CCCACAAGC/ZEN/ACAGAGA<br>CCAGGAG/3IABkFQ/-3'
NCC<br>NM_019415	5'-<br>CAGATCAACAGGATGGACGA<br>AG-3'	5'-<br>GCAATCATGTCCTCAAA<br>CCG-3'	5'-/56-FAM/TCCCGACAT/ZEN/CA<br>ACCAGA AGCCC/3IABkFQ/-3'
NDCBE<br>NM_021530.2	5'-<br>ATGGGAAGATGGTGAGGAAC-<br>3'	5'-<br>ACGTGCTCTTTTGGTCC<br>TG-3'	5'-/56-<br>FAM/AAGGTGCTG/ZEN/GAGACG<br>ATGAAGGG/3IABKFQ/-3'
NHE3<br>NM_N001081060.1	5'-<br>CCAAACAGCAAGAAATCCAG<br>G-3'	5'-<br>TCCCTCTATGGTGTCTT<br>CCTC-3'	5'-/56-FAM/CCA<br>ATTTC/ZEN/AGTTCACCCATCA<br>AGCCAC/3IABKFQ/-3'
NKCC2<br>NM_183354	5'-CAAGGAACATTACCAAGCC-<br>3'	5'-<br>CACCAGTTTGATTGAAC<br>TCC-3*	5'-/56-<br>FAM/CAAGAAAGA/ZEN/CGGCAAC<br>ATCAGCAGC/3IABKFQ/-3'
Pendrin<br>NM_011867	5'-<br>TGATGGAGGCAGAGATGAAT<br>G-3'	5'-<br>CAGAAAACACTGAGAGA<br>CTG-3'	5-/56-<br>FAM/ATGTTCAGG/ZEN/ATGAGGC<br>CATGCGTAG/3IABkFQ/-3'
Renin	TaqMan Gene Expression Assay (Applied Biosystems) REF# 4331182		
RPLP0<br>Mm.PT.58.43894205	5'-TTATA ACCCTGA<br>AGGCTCGAC-3'	5'-<br>CGCTTGTACCCATTGAT<br>GATG-3'	5'-/56-<br>FAM/AGGCCCTGC/ZEN/ACTCTCG<br>CTT/3IABkFQ/-3'

Disease Models & Mechanisms • Supplementary information

Table S2. Antibodies used for immunoblotting.

Name	Reference	Reference	Dilution
Rb anti-Claudin-10	Primary	Antibodies.com (ABIN3183935)	1:1000
Rb anti-Claudin-4	Primary	Invitrogen (36-4800)	1:1000
Rb anti-ENaC gamma	Primary	StressMarq Biosciences Inc (SPC-405)	1:5000
Direct-Blot HRP anti-β Actin	Primary	BioLegend (AB_2566701)	1:10000

Table S3. Baseline plasma characterization of WT and L919X Ae1 KI/KI mice.

Parameter	Units	WT (n=10)	L919X AE1 KI/KI (=10)	p-value
Na^+	mM	147.5 ± 0.563	146.9 ± 0.623	0.4327
Cl^-	mM	118.8 ± 0.964	119.6 ± 1.536	0.6644
K^+	mM	4.633 ± 0.167	4.610 ± 0.196	0.7029
HCO_3^-	mM	18.38 ± 0.563	19.95 ± 0.511	0.0930
pH		7.289 ± 0.002	7.249 ± 0.009	0.1186
TCO_2	mmHg	19.50 ± 0.749	21.30 ± 0.517	0.0636
BUN	mg/dl	22.70 ± 1.382	19.80 ± 0.987	0.1050
Glucose	mg/dl	181 ± 13.11	198.3 ±10.58	0.3181
HCT	%	36.80 ± 0.554	36 ± 0.789	0.4174
pCO_2	mmHg	38.99 ± 2.856	45.75 ± 1.595	0.0525
AnGAP	mM	14.6 ± 0.618	13.22 ± 1.325	0.1197
Hb	g/dl	12.53 ± 0.188	12.23 ± 0.269	0.3729
BEecf	mM	-8.100 ±0.781	-7.100 ± 0.504	0.2963

Data is presented as mean ± SEM. Two-tailed Student's T-Test or Mann-Whitney test used where appropriate.TCO_2, total carbon dioxide concentration including bicarbonate, dissolved carbonic acid, carbonate ion and CO2 combined with plasma proteins; BUN, blood urea nitrogen; HCT, hematocrit; AnGAP, anion gap; Hb, hemoglobin; BEecf, base excess of extracellular fluid.

Table S4. Baseline plasma characterization of WT and R607H Ae1 KI/KI mice.

Parameter	Units	WT (n=11)	R607H AE1 KI/KI (n=9)	p-value
Na^+	mM	146.4 ± 0.472	147.1 ± 0.586	0.3148
Cl^-	mM	118.2 ±1.110	118.5 ± 0.619	0.1409
**K^+**	**mM**	**4.5 ± 0.142**	**4.920 ± 0.077***	**0.0207**
HCO_3^-	mM	18.66 ±0.627	19.07 ±0.463	0.6142
pH		7.256 ± 0.014	7.258 ± 0.013	0.9102
TCO_2	mmHg	19.91 ± 0.653	20.20 ± 0.533	0.7370
**BUN**	**mg/dl**	**21.09 ± 1.351**	**23.60 ± 0.921***	**0.0438**
Glucose	mg/dl	196.3 ± 7.065	197.3 ± 12.31	0.9417
HCT	%	37.73 ± 1.221	38.60 ± 0.340	0.5178
pCO2	mmHg	42.05 ± 1.467	42.72 ± 1.410	0.7417
AnGAP	mM	13.27 ± 1.538	14.60 ± 0.600	0.9863
Hb	g/dl	12.82 ± 0.414	13.14 ± 0.117	0.4826
BEecf	mM	-8.545 ± 0.731	-8.100 ± 0.586	0.5666

Data is presented as mean ± SEM. Two-tailed Student's T-Test or Mann-Whitney test used where appropriate. ***Significant data is bolded.*** *TCO_2, total carbon dioxide concentration including bicarbonate, dissolved carbonic acid, carbonate ion and CO2 combined with plasma proteins; BUN, blood urea nitrogen; HCT, hematocrit; AnGAP, anion gap; Hb, hemoglobin; BEecf, base excess of extracellular fluid.*

Table S5. Plasma characterization of WT and L919X Ae1 KI/KI mice following 24 hours of salt restriction.

Parameter	Units	WT (n=10)	L919X AE1 KI/KI (n=10)	p-value
Na^+	mM	147.9 ± 0.690	147.7 ± 0.578	0.8268
Cl^-	mM	117.6 ± 0.897	120.9 ± 1.329	0.0543
K^+	mM	4.980 ± 0.158	5.180 ± 0.464	0.5661
HCO_3^-	mM	20.59 ± 0.613	20.40 ± 0.825	0.8554
pH		7.240 ± 0.021	7.248 ± 0.033	0.8469
TCO_2	mmHg	22.20 ± 0.593	21.80 ± 0.854	0.7048
BUN	mg/dl	30.10 ± 4.365	23.30 ± 0.989	0.1249
Glucose	mg/dl	164.5 ± 12.69	157.1 ± 9.719	0.6490
HCT	%	40.70 ± 0.667	38.90 ± 0.690	0.1152
pCO2	mmHg	47.95 ± 1.127	47.74 ± 3.411	0.9540
AnGAP	mM	14.90 ± 0.795	11.89 ± 1.369	0.0677
Hb	g/dl	13.84 ± 0.225	13.22 ± 0.237	0.0741
BEecf	mM	-6.900 ± 0.971	-6.800 ± 1.073	0.9457

Data is presented as mean ± SEM. Two-tailed Student's T-Test or Mann-Whitney test used where appropriate. ***Significant data is bolded.*** *TCO_2, total carbon dioxide concentration including bicarbonate, dissolved carbonic acid, carbonate ion and CO2 combined with plasma proteins; BUN, blood urea nitrogen; HCT, hematocrit; AnGAP, anion gap; Hb, hemoglobin; BEecf, base excess of extracellular fluid.*

Table S6. Plasma characterization of WT and R607H Ae1 KI/KI mice following 24 hours of salt restriction.

Parameter	Units	WT (n=12)	R607H AE1 KI/KI (n=14)	p-value
Na^+	mM	146.3 ± 0.890	148.1 ± 0.275	0.8166
Cl^-	mM	121.5 ± 2.062	122.9 ± 0.769	0.5196
**K^+**	**mM**	**4.475 ± 0.100**	**4.843 ± 0.100**	**0.0162**
HCO_3^-	mM	19.04 ± 0.965	18.39 ± 0.540	0.5437
pH		7.203 ± 0.021	7.215 ± 0.014	0.6389
TCO_2	mmHg	20.50 ± 1.004	19.86 ± 0.592	0.9305
BUN	mg/dl	23.75 ± 1.473	25.00 ± 1.267	0.5236
Glucose	mg/dl	154.5 ± 11.59	182.6 ± 9.245	0.0667
HCT	%	37.50 ± 2.183	37.86 ± 0.710	0.1096
pCO2	mmHg	48.33 ± 2.356	45.75 ± 1.901	0.3983
AnGAP	mM	10.25 ± 2.394	11.64 ± 0.608	0.1453
Hb	g/dl	12.76 ± 0.741	12.86 ± 0.246	0.0994
BEecf	mM	-9.000 ± 1.206	-9.357 ± 0.608	0.7847

Data is presented as mean ± SEM. Two-tailed Student's T-Test or Mann-Whitney test used where appropriate. ***Significant data is bolded.*** *TCO_2, total carbon dioxide concentration including bicarbonate, dissolved carbonic acid, carbonate ion and CO2 combined with plasma proteins; BUN, blood urea nitrogen; HCT, hematocrit; AnGAP, anion gap; Hb, hemoglobin; BEecf, base excess of extracellular fluid.*

Table S7. Plasma characterization of WT and L919X Ae1 KI/KI mice following salt depleted acid load.

Parameter	Units	WT (n=14)	L919X AE1 KI/KI (n=14)	p-value
K^+	mM	4.777 ± 0.154	4.669 ± 0.170	0.6430
HCO_3^-	mM	15.63 ± 0.940	13.88 ± 0.470	0.1164
pH		7.170 ± 0.030	7.131 ± 0.014	0.0683
**TCO_2**	**mmHg**	**17.62 ± 0.789**	**15.15 ± 0.504**	**0.0147**
**BUN**	**mg/dl**	**23.75 ± 0.954**	**33.62 ± 1.723**	**< 0.0001**
Glucose	mg/dl	148.9 ± 12.93	131.92 ± 5.054	0.2452
HCT	%	37.38 ± 1.274	37.46 ± 1.264	0.9713
pCO2	mmHg	42.84 ± 2.581	41.72 ± 1.632	0.7216
AnGAP	mM	13.33 ± 1.694	13.09 ± 0.948	0.9042
Hb	g/dl	12.70 ± 0.432	12.74 ± 0.430	0.9713
BEecf	mM	-12.93 ± 1.294	-15.31 ± 0.603	0.1169

Data is presented as mean ± SEM. Two-tailed Student's T-Test or Mann-Whitney test used where appropriate. ***Significant data is bolded.*** *TCO_2, total carbon dioxide concentration including bicarbonate, dissolved carbonic acid, carbonate ion and CO2 combined with plasma proteins; BUN, blood urea nitrogen; HCT, hematocrit; AnGAP, anion gap; Hb, hemoglobin; BEecf, base excess of extracellular fluid.*

Table S8. Plasma characterization of WT and R607H Ae1 KI/KI mice following salt depleted acid load.

Parameter	Units	WT (n=14)	R607H AE1 KI/KI (n = 18)	p-value
K^+	mM	4.557 ± 0.229	4.406 ± 0.185	0.6069
**HCO_3^-**	**mM**	**14.15 ± 0.921**	**11.64 ± 0.775**	**0.0448**
**pH**		**7.192 ± 0.025**	**7.085 ± 0.020**	**0.0016**
TCO_2	mmHg	15.29 ± 0.963	12.83 ± 0.797	0.0572
**BUN**	**mg/dl**	**22.50 ± 1.725**	**34.17 ± 3.618**	**0.0026**
**Glucose**	**mg/dl**	**163.2 ± 11.93**	**118.8 ± 7.284**	**0.0024**
HCT	%	34.00 ± 2.176	32.56 ± 2.297	0.8434
pCO2	mmHg	46.21 ± 1.429	38.56 ± 2.400	0.4397
AnGAP	mM	10.08 ± 2.065	13.43 ± 1.462	0.6004
**Hb**	**g/dl**	**11.56 ± 0.739**	**13.55 ± 0.189**	**0.0489**
**BEecf**	**mM**	**-14.07 ± 1.273**	**-18.22 ± 0.967**	**0.0129**

Data is presented as mean ± SEM. Two-tailed Student's T-Test or Mann-Whitney test used where appropriate. ***Significant data is bolded.*** *TCO_2, total carbon dioxide concentration including bicarbonate, dissolved carbonic acid, carbonate ion and CO2 combined with plasma proteins; BUN, blood urea nitrogen; HCT, hematocrit; AnGAP, anion gap; Hb, hemoglobin; BEecf, base excess of extracellular fluid.*
